# Evaluation of the Antioxidant Capacity of Fruit Juices by Two Original Analytical Methods

**DOI:** 10.3390/molecules28186672

**Published:** 2023-09-18

**Authors:** Michele Protti, Isacco Gualandi, Sergio Zappoli, Roberto Mandrioli, Laura Mercolini, Domenica Tonelli

**Affiliations:** 1 Department of Pharmacy and Biotechnology (FaBiT), Alma Mater Studiorum-University of Bologna, Via Belmeloro 6, 40126 Bologna, Italy; michele.protti2@unibo.it (M.P.); laura.mercolini@unibo.it (L.M.); 2 Department of Industrial Chemistry “Toso Montanari”, Alma Mater Studiorum-University of Bologna, Viale del Risorgimento 4, 40136 Bologna, Italy; isacco.gualandi2@unibo.it (I.G.); sergio.zappoli@unibo.it (S.Z.); 3 Department for Life Quality Studies (QuVi), Alma Mater Studiorum-University of Bologna, Corso d’Augusto 237, 47921 Rimini, Italy; roberto.mandrioli@unibo.it

**Keywords:** antioxidant capacity, hydroxyl radicals, Fenton’s reaction, hydrogen peroxide photolysis, HPLC with coulometric detection, polyphenol modified electrode

## Abstract

Two analytical methods previously developed by our groups were employed to estimate the antioxidant capacity of commercial fruit juices. The electrochemical method, which measures the scavenging activity of antioxidants towards OH radicals generated by both hydrogen peroxide photolysis and Fenton’s reaction, is based on the recovery of the cyclic voltametric response of the redox probe Ru(NH_3_)_6_^3+^ at a Glassy Carbon electrode modified with a thin film of an insulating polyphenol, in the presence of compounds with antioxidant properties. The values of the antioxidant capacity of the fruit juices are expressed as vitamin C equivalents/L. The chromatographic method is based on the generation of OH radicals via Fenton’s reaction in order to test the inhibition of their formation in the presence of antioxidant compounds by monitoring salicylate aromatic hydroxylation derivatives as markers of •OH production, by means of HPLC coupled to coulometric detection. The results are expressed as the percentage of inhibition of •OH production in the presence of the tested juice compared to the control sample. When OH radicals are produced by Fenton’s reaction, the antioxidant capacity of the juices, estimated by both methods, displays an analogous trend, confirming that they can be considered an alternative for measuring the ability of antioxidants to block OH radical formation.

## 1. Introduction

The recent shift from the traditional medical care approach to personalized medicine, which emphasizes the prevention rather than the cure of diseases, has raised awareness of how important lifestyles and the quality of nutrition are. In this context, antioxidants from fruits and vegetables are considered bioactive dietary compounds which can reduce oxidative stress, and epidemiological data have indicated an inversed correlation between the intake of fruits and vegetables, which are naturally rich in antioxidants, and the incidence of some illnesses such as cardiovascular or metabolic disorders and cancer [1]. Oxidative stress takes place when an unbalance occurs between chemically reactive oxidative species, i.e., reactive oxygen species (ROS), reactive nitrogen species (RNS), reactive sulfur species (RSS) and reactive carbonyl species (RCS), and the ability of the organism to counteract their action through its antioxidant protective systems, including both antioxidant enzymes and endogenous antioxidants [2].

From a nutritional point of view, an antioxidant is defined as any compound which, when present in low concentrations as compared to those of an oxidable substrate, is able to significantly delay or inhibit the oxidation of that substrate [2].

Therefore, the analysis of these compounds and the evaluation of the antioxidant activity/capacity of various foods and beverages have grown considerably in recent years [3]. Unfortunately, a common issue in this research field is how to measure such an activity/capacity and express the results. In fact, although a great effort has been made to standardize the analytical methods, their results are often lacking concordance, and that can be ascribed to the different mechanisms that antioxidants play and the different principles on which the various assays for measuring antioxidant capacity are based [4].

Among the most commonly employed assays for evaluating the antioxidant properties of food, we can cite DPPH, ABTS, Folin-Ciocalteau, CUPRAC, FRAP, ORAC and TRAP [4].

In 2015, Tonelli’s group proposed an electrochemical method (OH-RSC), which measured the scavenging activity of antioxidants towards OH radicals generated by hydrogen peroxide photolysis. The method was based on the recording of the cyclic voltametric response (CV) of a redox probe, Ru(NH_3_)_6_^3+^, at a Glassy Carbon electrode (GCE) modified with a thin film of an insulating polyphenol, after it had been exposed to OH radicals. In fact, the intensity of the recovered electrochemical signal was proportional to the extent of the polymer degradation, as the redox probe was fully unresponsive when the polyphenol film was just electrodeposited on the GCE. The antioxidant capacity (AOC) of some standard compounds and fruit juices estimated by the OH-RSC method was compared with that obtained using more standardized assays, like ABTS, DPPH and ORAC. The best correlation among the obtained results was noticed with the ORAC assay [5,6]. Later, the electrochemical method was applied to the analysis of the AOC of dried goji berries [7], which were also analyzed by an LC–MS/MS method to determine the concentration of 23 different analytes with well-known antioxidant properties. In that work, an extraction and a pretreatment procedure were also developed, and the overall analytical method was validated according to international guidelines. The conclusion was that there was a substantial agreement between the results obtained from the two investigations.

Mercolini’s group has recently developed and optimized an in vitro Fenton’s system to test the variation of •OH formation by monitoring salicylate aromatic hydroxylation derivatives as markers of •OH production, namely, 2,3- and 2,5-dihydroxybenzoic acid (2,3-DHBA and 2,5-DHBA) and catechol. Salicylic acid was chosen as the substrate because it easily undergoes radical hydroxylation, producing catechol, which originates from the aromatic radical addition of a hydroxyl group in position one, followed by decarboxylation, while the other two products are derived by the same addition in other activated positions (three and five). These molecules are electroactive due to the vicinal phenol groups (catechol) that can be easily oxidized or reduced [8], and the products can be detected by electrochemical detection (ED) with high sensitivity, particularly by coulometric detection thanks to the specific cell geometry allowing for consistent and extensive compound oxidation (or reduction) [9]. The target analytes were determined by HPLC coupled to coulometric detection using a fully validated method, which was then exploited to evaluate the antioxidant and/or prooxidant activity of various bioactive molecules by comparing hydroxylation products’ (and, thus, •OH) formation rates in Fenton’s reaction mixtures with and without bioactive molecules [10].

The aim of the present work was to analyze samples of fruit juices commercially available in the Italian market by means of the analytical methods proposed by Tonelli’s and Mercolini’s groups [6,9] to verify if they can be considered in some way equivalent in estimating the antioxidant capacity, thus providing an alternative yet reliable platform to be exploited in investigating both the antioxidant and prooxidant capacity of significant bioactive compounds, natural extracts and foods rich in chemically different compounds, also paving the way for more detailed studies on the antioxidant and prooxidant mechanism of action of in vivo complex systems.

## 2. Results and Discussion

In Table 1, the results obtained from the chromatographic method when applied to the reference standard Fenton’s reaction in terms of the mean peak area of the three hydroxylation products are reported.

**Figure 1:**
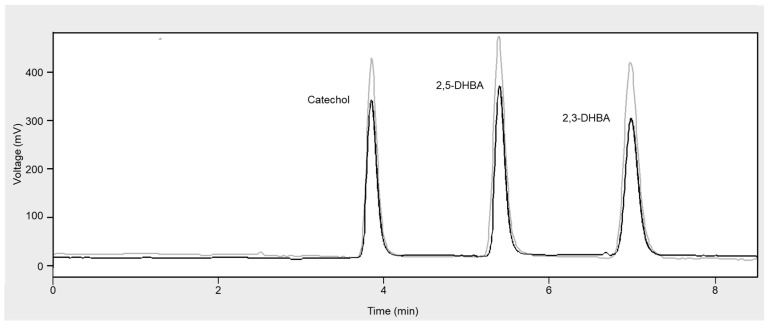
HPLC-ED chromatograms obtained from the analysis of salicylic acid hydroxylation products after carrying out a standard Fenton’s reaction (grey line) and in the presence of a fruit juice sample (black line).

Figure 1 reports typical HPLC-ED chromatograms obtained from the analysis of a reference Fenton’s reaction mixture alone and in the presence of a fruit juice sample.

In Table 2, the data obtained from the two analytical methods for all the analyzed juices are shown. As already stated above, the results from the electrochemical method are expressed as Vitamin C equivalents/L of the sample, whereas the results from the chromatographic method are expressed as the percentage of inhibition of •OH production in comparison to that of the control sample not containing the tested juice.

In fact, in such a case, a decrease in the production of catechol, 2,3-DHBA and 2,5-DHBA, as evidenced by the chromatograms in Figure 1, indicates a corresponding proportional decrease in •OH production, which is consequently related to the antioxidant capacity of the fruit juice under investigation.

Even if the results from the two methods are calculated in a different way, it is easy to draw some conclusions.

Taking into account the data coming from the electrochemical method, it is possible to say that the VCE values are significantly higher when the OH radicals are produced by the Fenton’s reaction than when the H_2_O_2_ photolysis is employed. That is explainable considering that, in the former case, the modified electrode estimates the ability of antioxidants to block OH radical formation, whereas in the latter, it estimates the ability to remove OH radicals. Moreover, the VCE indexes of the different juices from the photolytic production of OH radicals are quite close together since the maximum value (pomegranate juice, sample J) is 1.68 and the minimum (blueberries mix juice, sample F) is 0.25 mol/L, respectively. On the contrary, the data from the Fenton’s reaction allow for highlighting a net difference between some samples that result in possessing a very high AOC (samples K and J, corresponding to a goji berry and pomegranate juice, respectively, with values of 22.7 and 16.9 mol/L) and those that result in exhibiting a very low AOC (samples C and A, with a VCE less than 1). In addition, a very good reproducibility for the analytical measurement of real samples AOC was observed when the OH radicals were produced by Fenton’s reaction, as highlighted by the percentage RSD values.

By comparing the results coming from the two analytical methods, considering again the Fenton’s reaction as the source of OH radicals for the electrochemical one, it is possible to observe that three groups of samples can be identified; the first consists of two juices which possess a very high AOC, the second consists of four juices whose AOC can be considered intermediate and the third consists of five juices which display a lower AOC.

In particular, the juices belonging to the three groups are the same for both methods, with only the exception of samples B and D, which are exchanged between the second and the third group. Therefore, we can state that the trend of the AOC values for the tested samples is in good agreement when both the proposed methods are employed. To further corroborate this conclusion, the AOC of the samples was also determined by three analytical methods, commonly employed for food analysis, i.e., ABTS, DPPH and ORAC, according to an experimental procedure already published [5]. The relevant results are shown in Table 3. In such a case, they are expressed as mmol Vitamin C equivalents per L of the fruit juice, since ABTS, DPPH and ORAC generally provide lower values than the ones obtained from the electrochemical method, and, in particular, those from the DPPH assay are the lowest, as already pointed out [5], as it is based on the scavenging capacity of antioxidants towards a rather stable nitrogen radical.

Although the numerical values are different and depend on the employed analytical method, it is possible to highlight, again, three groups of samples displaying high, intermediate and low AOC values. In any case, the sample J is the most active, and the sample F is one of the least active, when analyzed by ORAC, DPPH and ABTS assays, and this result is in rather good agreement with the data displayed in Table 2, relevant to the two methods proposed here for the evaluation of the AOC of fruit juices. In conclusion, even if the number of analyzed samples is not particularly high, the electrochemical and chromatographic methods can be considered alternative approaches to estimate the antioxidant capacity of real samples since both measure the ability of antioxidants to block OH radical formation. This result is remarkable, as the OH radical formation and inhibition in the chromatographic method always occur in a solution, whereas in the electrochemical method, the OH radicals must arrive from the solution to the modified electrode and attack the polyphenol film in order to be quantified. In the latter case, it is more likely that other compounds present in the fruit juices can react with the OH radicals, and this fact can explain why the VCEs of the samples with the highest (K) and lowest value (A) are in a ratio greater than 30, whereas the inhibition percentage ratio between the sample displaying the highest inhibition and that with the lowest is about 1.5.

## 3. Materials and Methods

### 3.1. Reagents

All chemicals and reagents were of analytical grade and used as received. L-Ascorbic acid (vitamin C), hexa-aminoruthenium(III) chloride, hydrogen peroxide solution (30% *w*/*w*), FeSO_4_, catechol, 2,3-DHBA, 2,5-DHBA, salicylic acid, CuCl, NaCl, NaHCO_3_, H_3_PO_4_, triethylamine and HPLC-grade acetonitrile were bought from Merck Life Science. CH_3_COONa, phenol and NaOH were purchased from Carlo Erba (Milan, Italy). Acetic acid and sulfuric acid were bought from Baker. Ultrapure water, obtained from a Millipore Milli-Q system (Burlington, MA, USA), was used throughout the experiments.

### 3.2. Electrochemical Method

The experimental procedure for evaluating the AOC has been already published [5]. Here, it is only briefly summarized.

The GCE was cleaned until a mirror-like surface was obtained; then, it was modified with the polyphenol film by performing a potentiostatic electropolymerization of phenol (0.05 M) in 1 M sulfuric acid, applying a potential of +1.0 V for 60 s. An aqueous saturated calomel electrode and a Pt wire were used as the reference and counter electrode, respectively. To check if a well-adhered film had been deposited, a CV in 0.5 M acetate buffer, pH 4.6, containing the redox probe Ru(NH_3_)_6_^3+^ (5 × 10^−3^ M), was recorded (blank signal). The absence of the redox probe signal indicated a satisfactory deposition.

The AOC of fruit juices was evaluated by studying the degradation kinetics of the polyphenol film in the absence and in the presence of the food samples, generating the OH radicals either by hydrogen peroxide photolysis or by Fenton’s reaction triggered by Fe(II) ions. Depending on the type and concentration of the antioxidants present in the fruit juice, the time requested to induce the polymer degradation increases due to the protective action of such compounds.

The photolytic generation of OH radicals was performed using a 120 mL reactor containing a 0.01 M H_2_O_2_ solution, where the modified electrode was placed near the lamp operating at 254 nm (UV18F, Italquarz, with emission power of 17 W) using a homemade holder that ensured the reproducibility of the sensor position during irradiation. In order to obtain the calibration line for vitamin C and to estimate the AOC of the analyzed juices, proper amounts of the standard antioxidant or of the samples were added. After irradiation, the electrode was taken out from the reactor at prefixed times, rinsed with ultrapure water and put in acetate buffer, where the CV signal related to the Ru(NH_3_)_6_^3+^ redox system was again recorded. The total irradiation time was generally 30 min.

For the generation of OH radicals by Fenton’s reaction, 20 mL of a 0.05 M FeSO_4_ solution containing a proper amount of Vitamin C or the sample were poured in an electrochemical cell where the modified GCE had been inserted. In total, 800 µL of 10 M H_2_O_2_ solution were added to start the reaction. After 10 min, the electrode was taken out from the solution, rinsed with ultrapure water and put in acetate buffer, where the CV signal related to the Ru(NH_3_)_6_^3+^ redox system was recorded.

### 3.3. Chromatographic Method

#### 3.3.1. Fenton Reaction

The formation of •OH via Fenton chemistry was monitored by the quantitation of three salicylic acid oxidation products: catechol, 2,3-DHBA and 2,5-DHBA.

A standard Fenton’s reaction in the presence of copper was triggered by adding 7.5 µL of 10 mM aqueous CuCl solution and 4 µL of 30% H_2_O_2_ to 947.5 µL of pH 7.40 bicarbonate solution (25 mM NaCl, 6.25 mM NaHCO_3_). This mixture was pre-incubated for 10 min at room temperature; then, 41 µL of 3 mM salicylic acid aqueous solution was added, and the reaction was carried out for 2 min at room temperature. A volume of 200 µL of 4% phosphoric acid was added to stop the reaction. Immediately afterward, an aliquot of 100 μL of the solution was removed and transferred into a vial for HPLC-ED analysis.

#### 3.3.2. HPLC-ED Conditions

The chromatographic system consisted of a model PU-1580 HPLC pump from Jasco (Tokyo, Japan) coupled to an ESA (Chelmsford, Massachusetts, MA, USA) coulometric Coulochem III detector, equipped with a high-sensitivity analytical cell with two porous graphite working electrodes placed in series in the same cell. The reference electrodes were α-hydrogen/palladium, and the support electrodes were made of 501 stainless steel. In the analytical cell, potential 1 was set to −200 mV and potential 2 was set to +450 mV, with a range of 200 nA and a +1.00 V output. The coulometric detector was also equipped with a conditioning cell set to a potential of +50 mV. The chromatograms were acquired in oxidation mode. Electrodes were cleaned at the end of each working week in order to prolong their use time and to obtain reproducible results. They were kept at +900 mV for 2 min to restore them, thoroughly oxidizing the components potentially fouling the electrodes. A Waters Cortecs C18 column (100 mm × 2.1 mm, 2.7 μm) equipped with a Waters Cortecs C18 VanGuard guard column (5 × 2.1 mm, 2.7 μm) was used as the stationary phase. The mobile phase was a mixture of triethylammonium phosphate buffer (50 mM; pH 2.00) and acetonitrile (88/12, *v*/*v*). HPLC analysis was carried out in isocratic mode at a flow rate of 1.0 mL/min, using an injection volume of 50 μL. The data integration system was the DataApex (Petrzilkova, Prague, Czech Republic) Chromatography Station software (version CSW32 1.4).

### 3.4. Methods Application to Fruit Juice Samples

#### 3.4.1. Electrochemical Method

The 11 kinds of commonly consumed fruit juices were purchased from local markets in Bologna and were randomly selected based on their contents of vitamin C and the kind of fruit that is known to contain high concentrations of antioxidant compounds. In total, 50 mL aliquots of the juices were accurately drawn and centrifuged at 6000 rpm for 20 min.

Each supernatant was immediately analyzed after proper dilution with ultrapure water, typically using a dilution ratio of 1:1000. The values of AOC of the fruit juices were expressed, taking Vitamin C as a reference, to give rise to vitamin C equivalents (VCE). VCE indexes were calculated on the basis of the vitamin C calibration line. Analyses were carried out five times for each sample. The AOC values of the 11 fruit juices were also determined by means of three commonly used methods, i.e., ABTS, DPPH and ORAC, according to an already published procedure [5].

#### 3.4.2. Chromatographic Method

To test the inhibition or stimulation of the •OH formation of the tested juices, each sample was diluted 1:100 (V/V) with ultrapure water; then, an aliquot of 7.5 µL of the diluted sample was added to the reaction mixture, replacing an equal volume of bicarbonate solution. After being subjected to Fenton’s reaction in the presence of salicylic acid, the mixture was injected into the HPLC-ED system. The concentrations of the three analytes (catechol, 2,5-DHBA and 2,3-DHBA) were determined separately by interpolation on the respective calibration curves and then added together; the obtained sum was compared to the sum of the analyte concentrations obtained without any tested fruit juice. The result was expressed as the percentage of inhibition of •OH production in comparison to that of the control sample not containing the tested sample. Analyses were carried out five times for each sample.

## 4. Conclusions

The data coming from the analysis of 11 fruit juices by means of the two original analytical methods, previously developed and validated by our groups, for evaluating their antioxidant capacity are in good agreement, especially if both employ Fenton’s reaction as the source of OH radicals. In such a case, even if the AOC is calculated and expressed in a different way by the electrochemical and chromatographic methods, three groups of samples are identified, which are characterized by a high, intermediate and low value of antioxidant capacity.

Therefore, we can state that the two analytical approaches can be considered alternatives to estimate the antioxidant capacity of real samples, since both measure the ability of antioxidants to block OH radical formation. Furthermore, they can be obviously exploited to investigate both the antioxidant and prooxidant capacity of significant bioactive compounds, natural extracts and foods in general.

## Figures and Tables

**Table 1:** Method application to a reference standard Fenton’s reaction ^a^.

Analyte	Average Concentration (nM)	RSD%
Catechol	201	2
2,5-DHBA	151	3
2,3-DHBA	384	2
Sum	7.4 × 10^2^	2

^a^ n = 5.

**Table 2:** Method application to fruit juice samples.

Sample	Description	Analyte Concentration Sum ^a^ (μM)	Variation with Respect to Standard Fenton ^a^ ((C_sample_ − C_Fenton_)/C_Fenton_)	Vitamin C Equivalents OH-RSC ^a^ (mol/L) (Fenton)	Vitamin C Equivalents OH-RSC ^a^ (mol/L) (Photolysis)
A	Blue fruits mix	0.624 (1)	−0.15 (1 × 10)	0.70 (4)	0.26 (2 × 10)
B	Yellow fruits mix	0.62 (2)	−0.15 (1 × 10)	1.74 (1)	0.7 (1 × 10)
C	Violet fruits mix	0.62 (3)	−0.16 (2 × 10)	0.94 (2)	0.85 (7)
D	Berries and red fruits	0.60 (7)	−0.18 (3 × 10)	1.29 (1)	0.68 (9)
E	Red fruits mix	0.60 (5)	−0.19 (3 × 10)	2.40 (1)	1.02 (6)
F	Blueberries mix	0.63 (2)	−0.15 (1 × 10)	1.05 (2)	0.25 (2 × 10)
G	Red fruits mix	0.62 (2)	−0.15 (1 × 10)	1.49 (1)	0.31 (2 × 10)
H	Carrot bio-centrifuged juice	0.60 (5)	−0.18 (3 × 10)	2.48 (1)	0.40 (1 × 10)
I	Cranberry juice	0.61 (3)	−0.17 (2 × 10)	1.80 (1)	1.33 (4)
J	Pomegranate juice	0.56 (4)	−0.23 (2 × 10)	16.9 (1)	1.68 (2)
K	Goji juice and pulp	0.58 (5)	−0.21 (2 × 10)	22.7 (1)	0.65 (6)

^a^ Average (percentage relative standard deviation, RSD%), n = 5.

**Table 3:** Application of standard methods to fruit juice samples.

Sample	Vitamin C Equivalents ABTS ^a^ (mmol/L)	Vitamin C Equivalents DPPH ^a^ (mmol/L)	Vitamin C Equivalents (ORAC) ^a^ mmol/L
A	10.9 (6)	1.63 (6)	80.7 (1)
B	8.70 (6)	1.20 (8)	63.7 (1)
C	5.30 (1 × 10)	1.14 (9)	93.6 (1)
D	4.32 (1 × 10)	3.15 (6)	170.1 (1)
E	6.24 (8)	2.45 (8)	169.9 (1)
F	5.26 (9)	2.35 (4)	66.0 (1)
G	6.33 (8)	2.20 (4)	77.0 (1)
H	12.90 (3)	4.15 (9)	105.5 (1)
I	9.30 (5)	1.81 (6)	77.7 (1)
J	26.90 (2)	4.30 (7)	110.5 (1)
K	20.90 (2)	3.58 (8)	89.3 (1)

^a^ Average (RSD%), n = 5.

## Data Availability

The data presented in this study are available on request from the corresponding author.
